# Protective Effects of *Glycyrrhiza* Total Flavones on Liver Injury Induced by *Streptococcus agalactiae* in Tilapia (*Oreochromis niloticus*)

**DOI:** 10.3390/antibiotics11111648

**Published:** 2022-11-18

**Authors:** Jinliang Du, Liping Cao, Jiancao Gao, Rui Jia, Haojun Zhu, Zhijuan Nie, Bingwen Xi, Guojun Yin, Yuzhong Ma, Gangchun Xu

**Affiliations:** 1 Key Laboratory of Integrated Rice-Fish Farming Ecology, Ministry of Agriculture and Rural Affairs, Freshwater Fisheries Research Center, Chinese Academy of Fishery Sciences, Wuxi 214081, China; 2 College of Veterinary Medicine, Agricultural University of Hebei, Baoding 071001, China; 3 Wuxi Fisheries College, Nanjing Agricultural University, Wuxi 214081, China

**Keywords:** *Glycyrrhiza* total flavones, *Streptococcus agalactiae*, tilapia, oxidative stress

## Abstract

Clinical studies have confirmed that *Glycyrrhiza* total flavones (GTFs) have good anti-hepatic injury, but whether they have a good protective effect on anti-hepatic injury activity induced by *Streptococcus agalactiae* in tilapia (*Oreochromis niloticus*) is unknown. The aims of this study were to investigate the protective effects of *Glycyrrhiza* total flavones on liver injury induced by *S. agalactiae* (SA) and its underlying mechanism in fish. A total of 150 tilapia were randomly divided into five groups, each with three replicates containing 10 fish: normal control group, *S. agalactiae* infection group, and three *Glycyrrhiza* total flavone treatment groups (addition of 0.1, 0.5, or 1.0 g of GTF to 1 kg of feed). The normal control group was only fed with basic diet, after 60 d of feeding, and intraperitoneal injection of the same volume of normal saline (0.05 mL/10 g body weight); the *S. agalactiae* infection group was fed with basic diet, and the *S. agalactiae* solution was intraperitoneally injected after 60 d of feeding (0.05 mL/10 g body weight); the three GTF treatment groups were fed with a diet containing 0.1, 0.5, or 1.0 g/kg of GTF, and the *S. agalactiae* solution was intraperitoneally injected after 60 d of feeding (0.05 mL/10 g body weight). After 48 h injection, blood and liver tissues were collected to measure biochemical parameters and mRNA levels to evaluate the liver protection of GTFs. Compared with the control group, the serum levels of glutamic oxaloacetic transaminase (GOT), glutamic pyruvic transaminase (GPT), alkaline phosphatase (AKP) and glucose (GLU) in the streptococcal infection group increased significantly, while the levels of total antioxidant capacity (T-AOC), superoxide dismutase (SOD), catalase (CAT) and reduced glutathione (GSH) decreased significantly; observations of pathological sections showed obvious damage to the liver tissue structure in response to streptococcal infection. *S. agalactiae* can also cause fatty liver injury, affecting the function of fatty acid β-oxidation and biosynthesis in the liver of tilapia, and also causing damage to function of the immune system. The addition of GTFs to the diet could improve oxidative stress injury caused by *S. agalactiae* in tilapia liver tissue to different degrees, promote the β-oxidation of fatty acids in the liver, accelerate the lipid metabolism in the liver, and repair the damaged liver tissue. GTFs have a good protective effect on liver injury caused by streptococcus.

## 1. Introduction

Streptococcal disease is a common bacterial disease in fish farming, which mainly occurs in the seasons with a temperature above 20 degrees, and summer is the peak season for this disease. The incidence of the disease can reach 20–50%, even higher in some areas, and the mortality rate can reach 90% in some regions. The disease can affect many kinds of fish, especially tilapia. Researchers have found that a variety of bacteria can cause liver injury diseases, such as *Escherichia coli, Streptococcus, Brucella, Staphylococcus aureus* and so on [1,2,3,4]. The most harmful bacterial disease in tilapia is streptococcal disease. Fish bacteria are mainly from *Streptococcus* or *Lactococcus* genera and include several pathogenic species such as *Streptococcus iniae*, *Streptococcus agalactiae*, *Streptococcus paramammary*, and *Lactococcus grigneri* according to the original disease [5]. *S. agalactiae* was the main pathogenic bacteria affecting tilapia. Zhu et al. found that *S. agalactiae* caused serious liver damage to tilapia liver tissue, with disordered arrangement of hepatocytes and unclear cord-like structure [6]. Luo et al. studied the pathological study of *S. agalactiae* on tilapia niloticus, and the results showed that the hepatocytes of tilapia infected with *S. agalactiae* were severely denaturated and necrotic, the nuclei disappeared, and a large area of necrosis appeared [7]. Ao et al. studied the effects of *S. agalactiae* infection on the blood and hepatopancreas biochemical parameters of tilapia; the results showed that biochemical indexes of AKP, SOD and T-AOC in liver tissues were significantly changed, and the anatomy of tilapia hepatopanthus showed symptoms of swelling and necrosis [8].

At present, the infection route and pathogenesis of *Streptococcus* infection in fish are still unclear. Chen et al. believe that the pathogenesis process of Streptococcus infection in fish is mainly divided into four steps: colonization of host cells, evasion of host cell defense, proliferation in vivo and diffusion in vivo, which causes damage to fish tissues and organs. Capsular polysaccharide, β-hemolysin, Cyclic adenosine 3, 5′-monophosphate (cAMP) factor and C5α peptidase are important virulence factors in the process of infection [9]. It has been reported that a series of neurological symptoms, such as circuses in tilapia after infection, are mainly regulated by serotonin [10]. Another study reported that *S. agalactiae* can escape the immune defense of phagocytic cells and migrate with macrophages in the body to infect other tissues [11]. Li et al. also pointed out that Streptococcus can colonize the gastrointestinal lumen of tilapia and invade the internal body to infect the host [12].

Regarding the treatment of tilapia streptococcosis, antibiotic therapy is a commonly used method in aquaculture. Although antibiotics have a good therapeutic effect on Streptococcus, the excessive use of antibiotics can easily cause bacterial resistance and drug residues in tilapia. With the proposal of the concept of green ecological breeding, Chinese herbal medicine has been more and more widely used in the breeding process, and there have been a large number of reports on the antibacterial effect of Chinese herbal medicine. Licorice (*Glycyrrhiza uralensis* Fisch.) is a plant in the leguminous, licorice family. The root of licorice is usually the medicinal part, which has been used in many traditional Chinese prescriptions. Flavonoids are the main active components isolated from roots and rhizomes of licorice. It mainly contains six flavonoids, 5-(1,1-dimethylallyl)-3,4,4′-trihydroxy-2-methoxychalcone, licochalcone B, licochalcone A, echinatin, glycycoumarin and glyurallin B [13,14,15]. Wang et al. found that crude extract of licorice has a good antibacterial effect on *Streptococcus suis* [16]. Liu studied the antibacterial activity of licorice extract, and found that oleic acid, dehydrocyrrhizin D, glycyrrhetinic acid had a good inhibitory effect on *Streptococcus mutans, Escherichia coli, Bacillus subtilis* and other bacteria [17]. Recent studies have found that GTF have good anti-inflammatory, antibacterial and antiviral effects [18,19,20]. However, there has been no relevant report on whether GTFs have an antibacterial effect on tilapia Streptococcus.

In this study, Nile tilapia (*Oreochromis niloticus*) were used as the experimental object, and the tilapia were infected by intraperitoneal injection of *S. agalactiae*. The effective ingredient of Chinese herbal medicine, *Glycyrrhiza* total flavones, was added to the diet. The serum biochemical indicators, histopathological observation and expression changes of related genes were used to evaluate whether GTFs had an antibacterial effect on Streptococcus tilapia. In order to provide a theoretical basis for the development of anti-streptococcal drugs.

## 2. Materials and Methods

### 2.1. Test Materials

#### 2.1.1. Ethics Statement

This experiment was approved by the Institutional Animal Care and Use Committee (IACUC) of the Chinese Academy of Fishery Science.

#### 2.1.2. Fish

Tilapia (*Oreochromis niloticus*) were obtained from the Freshwater Fisheries Research Center of the Chinese Academy of Fishery Science, Wuxi, China. The fish were healthy with an intact body surface and normal swimming posture, and the average body weight was 20 ± 1 g. After being retrieved from the fishery, the fish were kept in a circulating water system for 1 week prior to the experiment [21,22,23], the water temperature was set at 28 ± 1 °C; pH 6.7–7.3; dissolved oxygen, >6 mg/L, and fish were fed on a commercial diet purchased from Tongwei Group Co., Ltd. (Chengdu, China) twice each day.

#### 2.1.3. Reagents and Drugs

*Glycyrrhiza* total flavones (S25400, UV ≥ 98%) were purchased from Shanghai Yuanye Biotechnology Co., Ltd. (Shanghai, China). Glutamateruvate transaminase (GPT), glutamate oxalate transaminase (GOT), alkaline phosphatase (AKP), malondialdehyde (MDA), total antioxidant capacity (T-AOC), reduced glutathione (GSH) and superoxide dismutase (SOD) were purchased from Nanjing Jiancheng Haihao Bioengineering Co., Ltd. (Jiangsu, China). The reagents required for quantitative real-time PCR (qRT-PCR) were purchased from Takara (Dalian, China).

#### 2.1.4. Resuscitation and Culture of *S. agalactiae*

*S. agalactiae* (No. 619) was used in this study, which was collected by Dr. Xi from Laboratory of Aquatic Diseases and Feed, Freshwater Fisheries Research Center, Chinese Academy of Fishery Sciences. The *S. agalactiae* was resuscitated in brain-heart infusion via incubating overnight at 28 °C under 150 rpm. A single colony was selected for Gram staining to detect the purity of the culture. After identification, a single colony was inoculated into brain-heart liquid media and incubated for 24 h, then the cultured bacterial liquid was centrifuged at 6000 r/min for 5 min, the supernatant was discarded and the bacterial precipitate was added to sterilized physiological saline, and the obtained bacteria solution could be used for subsequent tests. 

### 2.2. Experimental Design and Parameter Measurement

#### 2.2.1. Test Groups

A total of 150 healthy tilapia with body weight of about 20 g were randomly divided into 5 treatment groups: a normal control group, a streptococcal injection group and 3 GTF treatment groups (addition of 0.1, 0.5, or 1.0 g/kg of total flavones to the diet), with three replicates in each group and 10 fish in each replicate. The fish were fed twice a day at 4% of their body weight for 60 days. The injection concentration was based on our preliminary results (LD_50_ 1.69 × 10^8^ CFU/mL). In the treatment group, the tilapia were injected with *S. agalactiae* at the concentration of 60% 96 h LD_50_. The normal control group and Streptococcal injection group were fed with the basal diet (31.6% crude protein, 4.6% crude lipid, 6.4% crude ash), and the three GTF-treated groups were fed with the basal diet supplemented with GTF at doses of 0.1, 0.5, and 1.0 g/kg diet, respectively. After 60 days feeding, the fish in the Streptococcal injection treatment group and GTF-treated groups were injected intraperitoneally (i.p.) with *S. agalactiae* (0.05 mL/10 g body weight), while the fish in the normal control group were administrated with the same volume saline (0.05 mL/10 g body weight). 

#### 2.2.2. Sample Collection and Processing

At 48 h after injection, the tilapia were anesthetized with 3-Aminobenzoic acid ethyl ester methanesulfonate (MS-222) [24,25,26]. The blood and liver tissues were collected from each treatment (10 fish/group) to investigate the protective effects of GTF against oxidative damage. The blood was collected by tail vein syringe, centrifuged at 5000 rpm for 10 min in a 4-degree centrifuge, and the separated serum was transferred to a 1.5 mL centrifuge tube. The serum and liver tissues were placed in a −80 degree refrigerator for storage.

#### 2.2.3. Measurement of Biochemical Indexes

The levels of glutamic oxaloacetic transaminase (GOT), glutamic pyruvic transaminase (GPT), alkaline phosphatase (AKP) and glucose (GLU) in the streptococcal infection group increased significantly, while the levels of total antioxidant capacity (T-AOC), superoxide dismutase (SOD), catalase (CAT) and reduced glutathione (GSH) in the serum were determined by kits from the Nanjing Jiancheng Bioengineering Institute (Nanjing, China). GPT and GOT were expressed as IU/L, AKP as U/L, SOD as U/mL, T-AOC as units/mL, CAT as U/L, GLU as mmol/L, and GSH as μmol/gprot. 

#### 2.2.4. Growth Performance Indicators

Feeding was stopped the day before the end of the experiment, and each fish was weighed after anesthesia (MS-222). The calculation formulas are as follows:

Weight gain rate, WGR, % = (W1 − W0)/W1 × 100%;

Specific growth rate, SGR, % = (lnW1 − lnW0)/T × 100%.

where W0 is the initial average body weight (g), W1 is the final average body weight (g), T is the farming time (d) of the experiment.

#### 2.2.5. Preparation and Observation of Liver Slices

The collected liver tissues of each group were fixed in Bouin’s fixative solution for 48 h, and paraffin tissue sections were prepared. The effect of GTF against *S. agalactiae* on liver tissue injury of tilapia was observed using HE staining.

#### 2.2.6. Real-Time Quantitative RT-PCR

An appropriate amount of tilapia liver tissue was taken, and total RNA was extracted from liver tissue using RNAiso Plus reagent. The operation process was carried out according to the instructions. The purity of RNA was analyzed by absorbance analysis, and the quality of total RNA extraction was evaluated by the OD260/OD280 ratio between 1.7 and 2.1. At the end of the RNA concentration determination, cDNA was synthesized by reverse transcription reaction using a reverse transcription kit and stored in a −80 degree refrigerator for subsequent experiments. 

Real-time PCR was performed using TB Green^®^ Premix Ex Taq™ II kit (Takara Bio Inc., Dalian, China). The reaction system was 25 μL, TB Green Premix Ex Taq II (2X) 12.5 μL, 1 μL each of upstream and downstream primers, and 2 μL of cDNA template. Sterilized water 8.5 μL. A two-step PCR amplification procedure was used with specific reaction conditions as follows: one cycle at 95 °C for 30 s, followed by 40 cycles of 95 °C for 5 s and 60 °C for 30 s. The relative expression of target genes was determined by 2^−ΔΔCt^ method [27]. The primer of the target gene is shown in the figure below (Table 1).

#### 2.2.7. Statistical Processing

SPSS22.0 software was used to analyze all data, and the final results were expressed as mean ± standard error of the mean (SEM). One-Way ANOVA test was used to analyze the significance of differences between the data. * *p* < 0.05 was considered as significant difference, and ** *p* < 0.01 was considered as extremely significant difference.

## 3. Results

### 3.1. Effects of GTF on Growth Performance of Tilapia

As shown in Table 2, after 60 d of feeding, the relative weight gain rate and specific growth rate of tilapia were not significantly affected by the dietary GTF level compared with the control group (0 g/kg) (*p* > 0.05).

### 3.2. Effect of GTF on Liver Morphology of Tilapia

**Figure 1:**
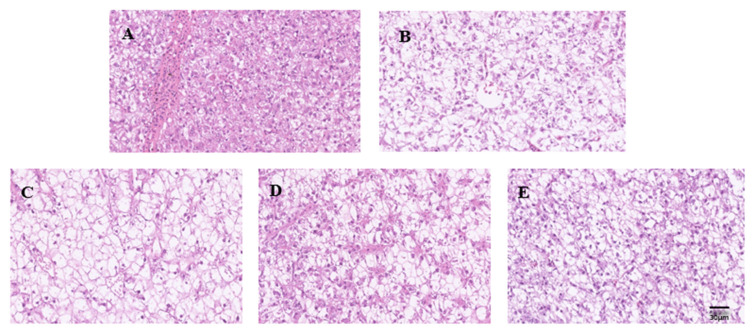
Effects of GTF on morphological changes of liver tissue of tilapia at different concentrations (bar = 30 μm). (**A**): Control group; (**B**): SA infection group; (**C**): 0.1 g/kg GTF treatment group; (**D**): 0.5 g/kg GTF treatment group; (**E**): 0.1 g/kg GTF treatment group.

As shown in Figure 1, the nucleus of the liver tissue in the normal group was clearly visible, and the thickness of the cords was uniform; in the SA infection group, some nuclei dissolved and disappeared, and there were round vacuoles with different sizes and uneven distribution in hepatocyte plasma, which showed reticular or transparent shape, and the staining became light. In addition, 1.0 g/kg GTF had a good protective effect against liver tissue damage caused by *S. agalactiae*, and could repair damaged hepatocytes.

### 3.3. Effects of GTF on Serum Biochemical Indices of Tilapia

**Figure 2:**
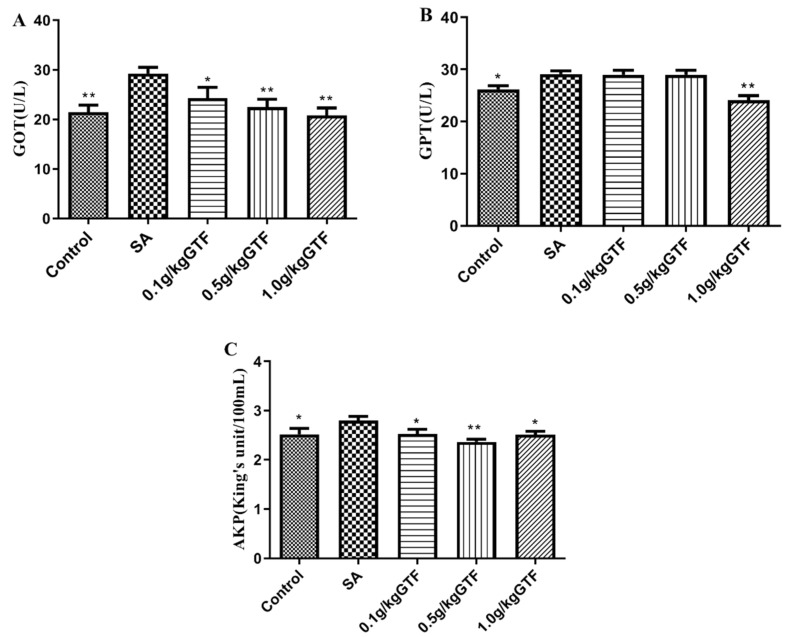
Effects of GTF on GOT (**A**), GPT (**B**) and AKP (**C**) activities in the serum of tilapia. Values are expressed as the mean ± SEM (*n* = 10). * *p* < 0.05; ** *p* < 0.01, compared with the control group.

As shown in Figure 2, at 48 h after *S. agalactiae* infection, the activities of GOT, GPT, and AKP in the serum increased significantly (*p <* 0.01 or *p <* 0.05) compared to the normal control group. The addition of 1.0 g/kg of GTF significantly decreased the activities of GOT, GPT, and AKP (*p* < 0.01).

### 3.4. Effects of GTF on Antioxidant System of Tilapia

**Figure 3:**
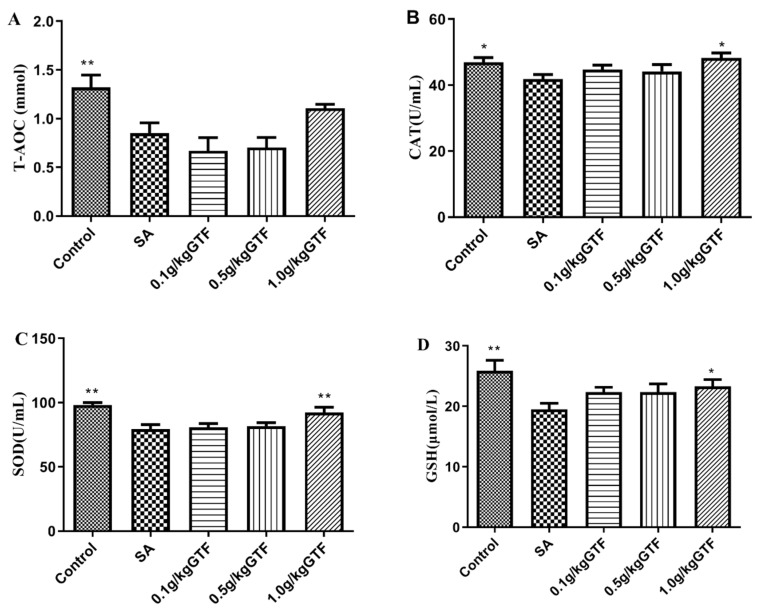
Effects of GTF on the activities of T-AOC (**A**), CAT (**B**), SOD (**C**) and GSH (**D**) in the serum of tilapia. Values are expressed as the mean ± SEM (*n* = 10). * *p* < 0.05; ** *p* < 0.01, compared with the streptococcal injection group.

As shown in Figure 3, after 48 h of *S. agalactiae* infection, the level of T-AOC, SOD, CAT, and GSH in serum were significantly decreased compared to the normal control group (*p* < 0.05 or *p* < 0.01). The addition of 1.0 g/kg of GTF could significantly increase the levels of antioxidant enzymes in serum, and alleviated the damage of streptococcus to tilapia.

### 3.5. Effects of GTF on Glucose Metabolism Indexes of Tilapia

**Figure 4:**
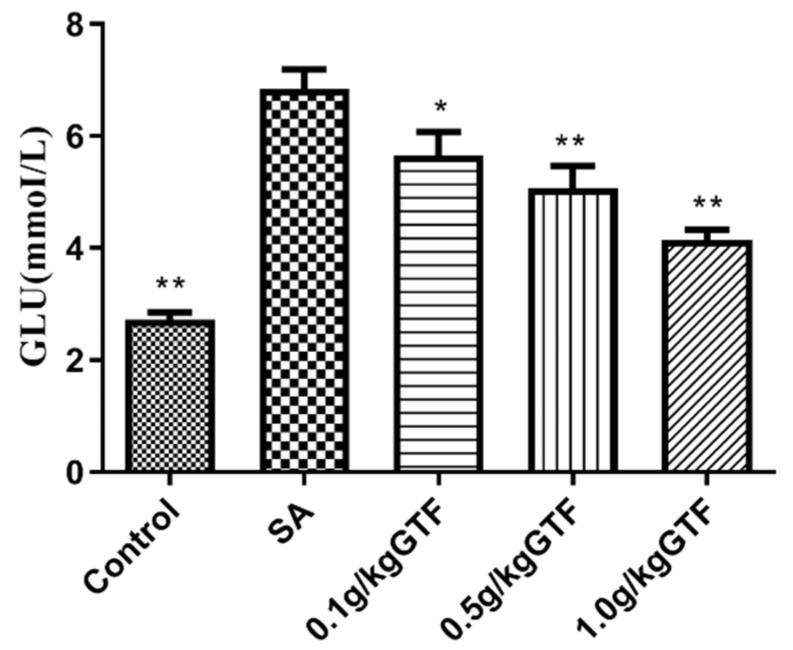
Effects of GTF on GLU level in the serum of tilapia. Values are expressed as the mean ± SEM (*n* = 10). * *p* < 0.05; ** *p* < 0.01, compared with the control group.

After 48 h, *S. agalactiae* infection significantly increased the content of GLU in serum (Figure 4; *p <* 0.01). However, 0.5 and 1.0 g/kg of GTF could significantly decrease the content of GLU in serum; the results showed that GTF could significantly improve the abnormal glucose metabolism caused by *S. agalactiae*.

### 3.6. Effects of GTF on β-Oxidation-Related Gene Rxpression in Tilapia

**Figure 5:**
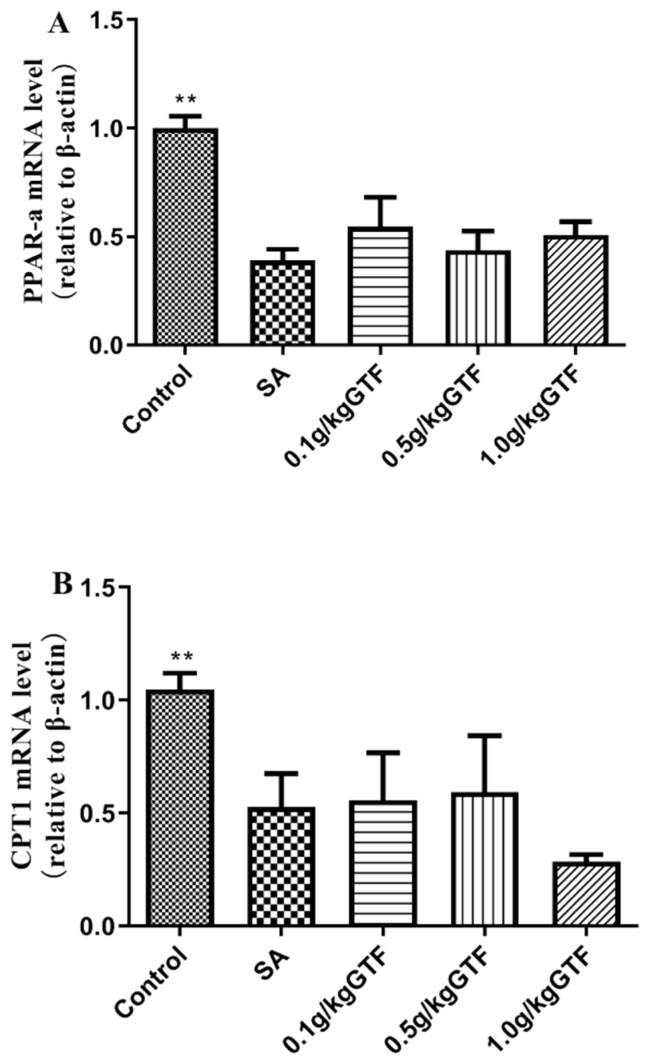

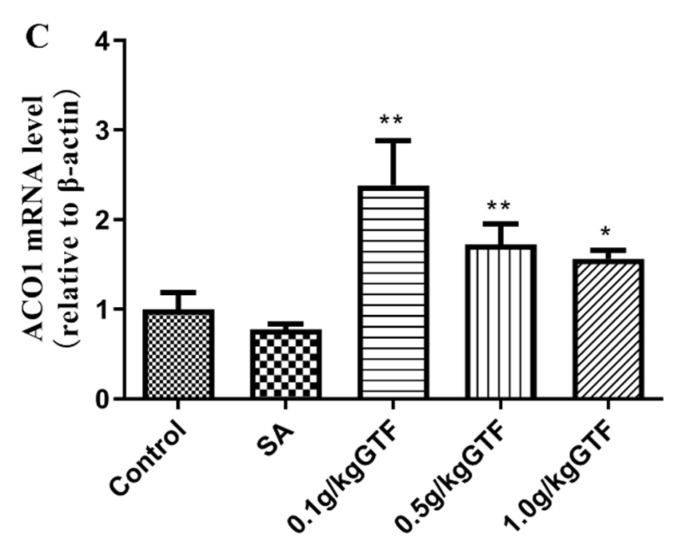
Effects of GTF on the mRNA levels of PPARa (**A**), CPT1 (**B**) and ACO1 (**C**) in the liver of tilapia. Values are expressed as mean ± SEM (*n* = 10). * *p* < 0.05 and ** *p* < 0.01 compared with streptococcal injection group.

As shown in Figure 5, the mRNA levels of carnitine palmitoyl transferase 1(CPT1) and peroxisome proliferator-activated receptor ɑ (PPARa) in the liver were significantly down-regulated with prolonged *S. agalactiae* infection time (*p* < 0.01); the expression of ACC oxidase gene (ACO1) gene was down-regulated, but the difference was not significant (*p* > 0.05). The addition of 0.1, 0.5, or 1.0 g/kg of GTF to the diet, the expression levels of the three genes in each concentration group could be increased to varying degrees, indicating that GTF had a certain alleviation effect on the oxidative damage of tilapia liver tissue caused by *S. agalactiae*. 

### 3.7. Effects of GTF on Expression of Genes Related to Lipid Droplet Formation in Tilapia

**Figure 6:**
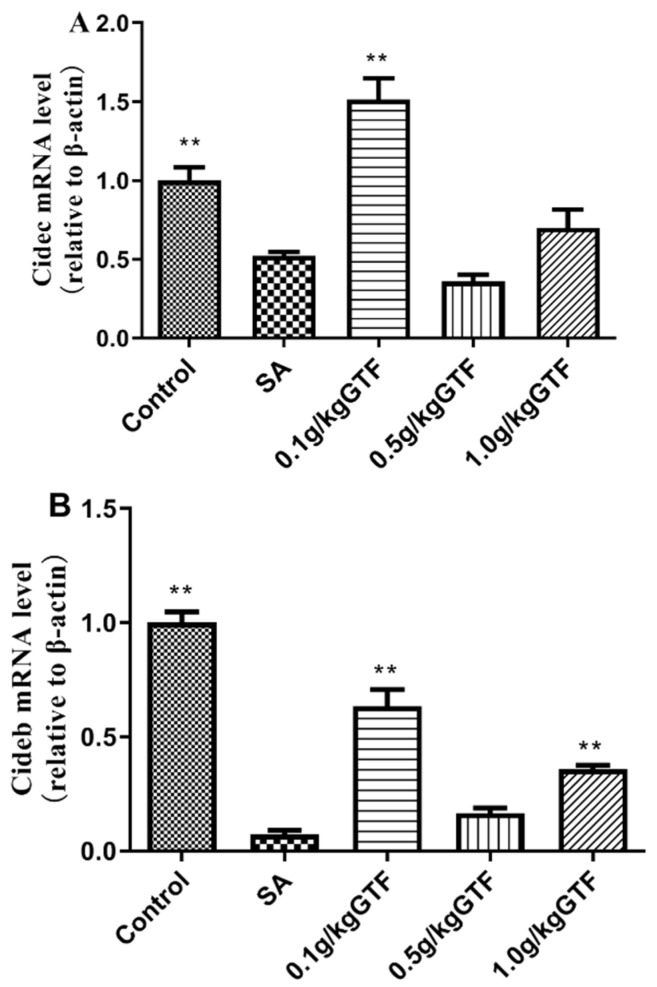
Effects of GTF on the mRNA levels of Cidec (**A**) and Cideb (**B**) in the liver of tilapia. Values are expressed as mean ± SEM (*n* = 10). ** *p* < 0.01 compared with streptococcal injection group.

As shown in Figure 6, the expression levels of cell-death-induced DFFA-like effector B(Cideb) and cell-death-induced DFFA-like effector C (Cidec) genes were significantly down-regulated in the livers of tilapia infected with *S. agalactiae* compared to the normal control group (*p* < 0.01). The addition of 0.1 g/kg of GTF could significantly up-regulate the expression level of Cidec mRNA (*p* < 0.01) and the addition of 0.1 or 1.0 g/kg of GTF could significantly up-regulate the expression level of Cideb mRNA (*p* < 0.01). This indicated that GTF could promote lipid metabolism in the liver.

### 3.8. Effects of GTF on Genes Related to Fatty Acid Formation in Tilapia

**Figure 7:**
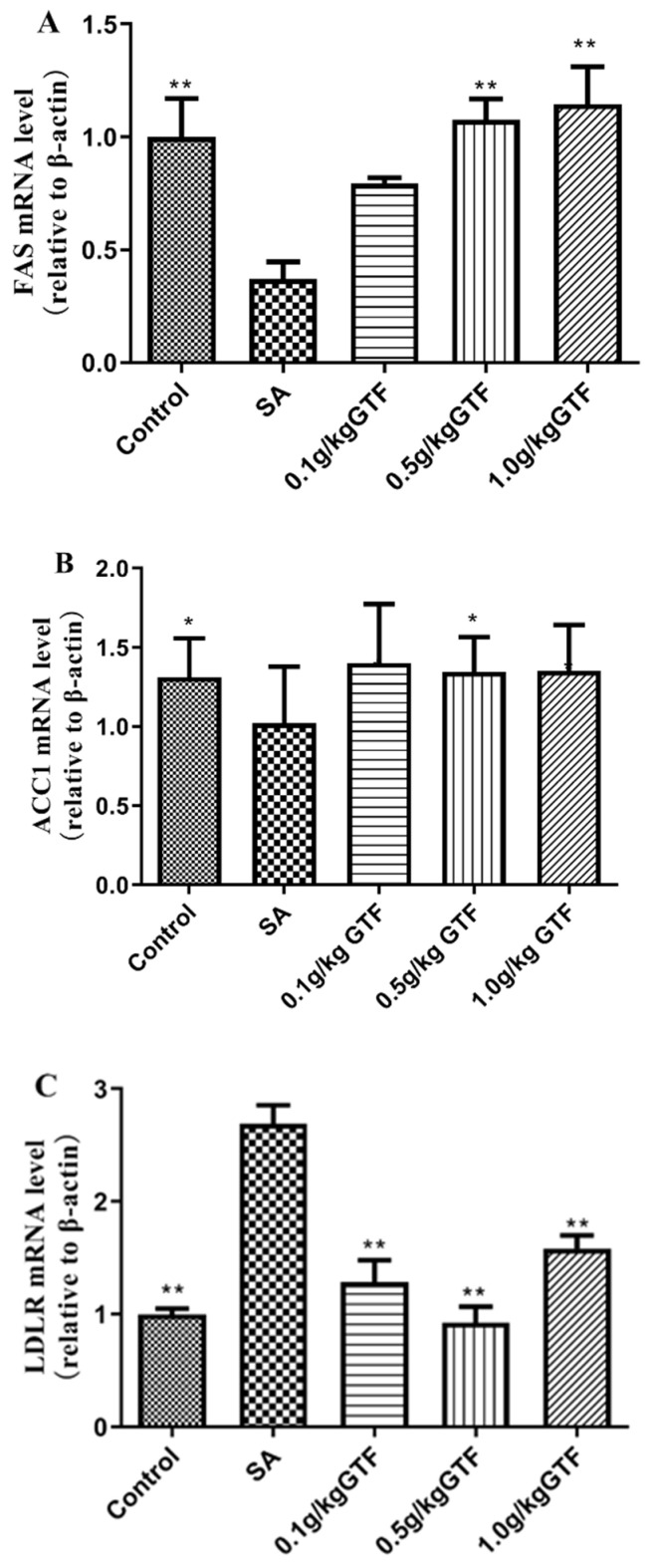
Effects of GTF on the mRNA levels of FAS (**A**), ACC1 (**B**) and LDLR (**C**) in the liver of tilapia. Values are expressed as mean ± SEM (*n* = 10). * *p* < 0.05 and ** *p* < 0.01 compared with streptococcal injection group.

As shown in Figure 7, the mRNA levels of acetylCoA carboxylase (ACC1) and fatty acid synthetase (FAS) in tilapia infected with *S. agalactiae* were significantly down-regulated (*p* < 0.01); the expression level of low-density lipoprotein receptor (LDLR) gene was significantly up-regulated compared to the normal control group (*p* < 0.01). In terms of the addition of 0.1, 0.5, or 1.0 g/kg of GTF to the diet, 0.5 g/kg GTF significantly up-regulated the expression levels of ACC1 and FAS genes in liver tissue, and addition of the 0.1 g/kg, 0.5 g/kg and 1.0 g/kg concentrations could significantly inhibit the expression levels of LDLR gene in liver tissue. 

### 3.9. Effects of GTF on the Expression of HSP70 and IgM Genes in Tilapia Liver Tissue

**Figure 8:**
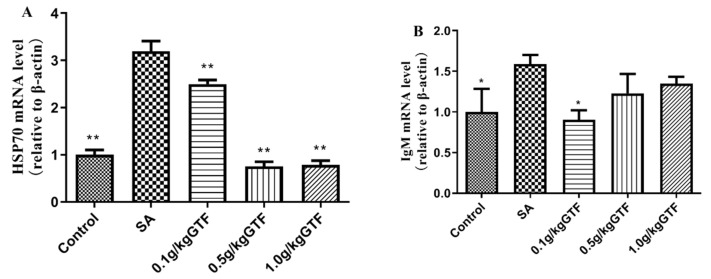
Effects of GTF on the mRNA levels of HSP70 (**A**) and IgM (**B**) in the liver of tilapia. Values are expressed as mean ± SEM (*n* = 10). * *p* < 0.05 and ** *p* < 0.01 compared with streptococcal injection group.

As shown in Figure 8, the expression levels of heat stress protein 70 (HSP70) and immunoglobulin M (IgM) genes were significantly up-regulated compared to the normal control group (*p* < 0.05 or *p* < 0.01). Addition of 0.1, 0.5, or 1.0 g/kg of GTF to the diet significantly down-regulated the HSP70 gene expression level (*p <* 0.01), and the addition of 0.1 g/kg of GTF could significantly down-regulate the IgM gene expression level (*p <* 0.05).

## 4. Discussion

Streptococcal disease is one of the main diseases that threaten tilapia culture, which has the characteristics of rapid infection, strong pathogenicity and high mortality. The disease occurs mostly from March to November, and can be transmitted through fish contact, feed or water environment. As previously reported, the most effective treatment of *Staphylococcus* diseases are connected by the use of such antibiotics as neomycin, enrofloxacin and florfenicol [28,29]. However, a large number of experimental studies have shown that antibiotics only have certain therapeutic significance for tilapia in the early stages of disease. Long-term use of antibiotics can not only cause the emergence of drug-resistant strains, but also can easily cause drug residues [30]. As a natural medicine, Chinese herbal medicine has the advantages of green environmental protection and no residue, and has been widely used in human beings and mammals, but the application research of aquatic animals is relatively small. In this study, the GTF were mainly used as feed additives to study whether it has a therapeutic effect on tilapia *S. agalactiae*, explore its specific anti-infection mechanism, and provide a new idea and method for the development of anti-Streptococcus infection drugs.

GOT, GPT and AKP are commonly used indicators to evaluate liver function [31]. After liver damage, the increased permeability of liver cell membrane will increase the release of GOT, GPT and AKP into the blood [32]. In the present study, the increased activities of GOT, GPT, and AKP enzymes confirmed that Streptococcus can cause liver tissue injury in tilapia. Qiang et al. studied the effects of *S. iniae* on biochemical indexes of different tilapia species, finding that *S. iniae* caused an increase in the activities of GOT, GPT, AKP and other enzymes in serum [33], which was consistent with the results of this study. After the intervention of GTF, 1.0 g/kg GTF could effectively reduce the enzyme activities of GPT, GSH and AKP in serum, which indicated that GTF could alleviate the liver tissue damage of tilapia caused by Streptococcus.

T-AOC, CAT, SOD and GSH are important components of the body’s antioxidant defense system, which play a crucial role in maintaining the dynamic balance between oxidation and antioxidant [34]. Studies have shown that the GTF is a natural free radical scavenger with good antioxidant effect [35,36]. Du et al. found that GTF had a good protective effect on tilapia liver injury caused by a high-fat diet, and could significantly improve GSH activity, SOD activity and T-AOC level in liver tissue [37]. Liu et al. found that the addition of licorice flavonoids in diets has a good therapeutic effect on the oxidative damage caused by ethanol in mice, which can improve the antioxidant capacity of the body, and increase the T-AOC and glutathione peroxidase (GSH-Px) levels in blood and liver tissues of mice [38]. Li et al. found that GTF could increase CAT levels in mice with myocardial injury caused by strenuous exercise [39]. The results of this experiment were similar to previous studies. Compared with the blank control group, the levels of T-AOC, CAT, SOD and GSH in serum of tilapia were significantly increased after adding GTF to the diet, indicating that GTF could improve the antioxidant capacity of tilapia and alleviate the damage of streptococcus to the antioxidant enzyme system of tilapia.

Peroxisome proliferator-activated receptor ɑ (PPARa) is a nuclear receptor protein that is abundant in the liver [40]. Studies have shown that PPARa is widely involved in energy metabolism, oxidative stress, inflammation and other physiological reactions in the body [41]. Carnitine palmitoyl transferase 1 (CPT1) exists in the outer membrane of mitochondria and is a rate-limiting enzyme of fatty acid oxidation, which plays an important role in energy metabolism [42,43,44]. Existing studies have proved that inhibition of fatty acid oxidation can effectively control blood glucose levels in the body [45,46]. Acyl-coenzyme A oxidase 1(ACO1) is a key enzyme in the oxidation of fatty acid β, which plays an important role in the decomposition of fatty acids [47]. The results of this study showed that the expression levels of PPARa, CPT1, and ACO1 genes were all down-regulated, indicating that streptococcus caused an impairment in the process of fatty acid β oxidation in tilapia, which affected the catabolism of fatty acids and resulted in an increase of glucose content in serum. After adding GTF to the diet, the expression levels of PPARa, CPT1 and ACO1 genes were increased to different degrees, and the glucose content in serum was decreased, indicating that GTF could promote the β-oxidation of fatty acids in the liver tissue of tilapia, protect hepatocytes, and alleviate the liver tissue damage caused by streptococcus.

A large number of experimental studies have confirmed that the cell-death-inducing DNA-fragmentation-factor (DFF45)-like effector (cide) family is involved in intracellular energy metabolism and is an important regulator in the body [48,49]. The cide family consists of three family members: cidea, cideb and cidec. Cideb mainly exists in liver tissue, and cideb plays an important role in cell apoptosis [50]; Chen et al. have also reported that cideb is involved in the process of fat metabolism in the liver [51]. Cidec mainly exists in adipose tissue, heart, intestine and other tissues, and can also be expressed in liver tissue. Li et al. have reported that cidec plays an important role in lipid metabolism, and abnormal cidec expression can lead to lipid metabolism disorders [52]. The function of cidec was also reported by foreign scholars, and it was found that cidec expression induced the apoptosis process of cells [53]. In this study, the expression of cidec and cideb genes was significantly down-regulated, indicating that Streptococcus affected the expression of the cide family in liver tissue, resulting in abnormal liver metabolic function and further liver tissue injury. The addition of GTF to the diet could increase the expression levels of cidec and cideb mRNA in liver tissue to different degrees, indicating that GTF could repair damaged liver tissues and promote liver lipid metabolism. 

FAS mainly exists in cytoplasm and is a fat metabolism enzyme with high expression in liver tissue. The amount of fatty acid synthetase plays an important role in the process of body fat deposition in animals. Some studies have reported that fatty acid synthetase is associated with the occurrence and development of diseases. Jia Rui et al. found that feeding tilapia with a high-fat diet for 60 days resulted in a significant decrease in FAS gene expression in the liver tissue of tilapia [54]. Zheng et al. found that the combination of theophylline and tea polyphenols could significantly reduce the expression of FAS gene in mouse liver tissue [55]. Acetyl-coa carboxylase (ACC) plays a major regulatory role in the process of fatty acid synthesis, which can regulate the oxidation of fatty acids [56]. Acc is highly expressed in the liver, and its activity can directly affect the fat metabolism of liver tissue. Qian et al. found that starvation stress could cause a significant decrease in the expression of ACC gene in the liver and muscle tissues of large yellow croaker [57]. Low-density lipoprotein receptor (LDLR) is a kind of membrane glycoprotein, which widely exists in various kinds of cells and tissues, plays an important role in lipoprotein metabolism. A large number of studies have shown that the abnormal blood lipid level in the body is closely related to the occurrence of many diseases. When studying the effect of bacterial lipopolysaccharides on LDLR gene expression in macrophages, Ye Qiang et al. found that with the increase of lipopolysaccharide concentration, LDLR gene expression level in macrophages was up-regulated in a concentration-dependent manner [58]. In this study, after streptococcal infection, the expression level of fatty acid synthetase gene in liver tissue was significantly down-regulated, while the expression level of low-density lipoprotein receptor gene in liver tissue was significantly up-regulated. The results of this experiment were similar to those of previous studies, indicating that *S. agalactiae* affected the normal lipid metabolism in the liver tissue of tilapia, causing lipid substances to accumulate in the liver tissue of tilapia, and then led to damage to the liver tissue. After adding GTF to the diet, three different concentrations of GTF can improve the damage of liver tissue of tilapia by *S. agalactiae* to different degrees, indicating that Chinese herbal medicine can regulate lipid metabolism and correct the disorder of lipid metabolism in liver tissue.

Heat stress protein 70 (HSP70) is one of the important members of the heat shock protein family, which plays a role in synergistic immunity and widely exists in animals, plants and microorganisms [59]. The level of HSP70 in normal cells is relatively low. When the cells are stimulated by external factors, the level of HSP70 will increase significantly, bind to the denatured proteins in the cells and degrade, thus playing a role in protecting the cells. Liu et al. studied the mechanism of HSP70 in carbon tetrachloride induced acute liver tissue injury in mice and found that the expression of HSP70 protein was significantly increased [60]; Ma et al. found that the expression of HSP70 in the liver tissue of mice induced by acetaminophen was significantly increased at 24 h [61]. IgM is an important component of the fish immune system, which plays an important role in resisting the stimulation of external pathogens such as pathogenic bacteria and other pathogens. Liu et al. studied the changes of immunoglobulin when carbon tetrachloride damaged the liver cells of Jian-carp and found that the content of IgM in the cells increased significantly [62]; Guo et al. found that lead exposure caused damage to the immune system of crucian carp (*Carassius auratus*), resulting in a significant increase in serum IgM level [63]. The results of this study were similar to previous studies; the expression levels of HSP70 and IgM genes in the streptococcal infection group were significantly increased compared to the blank control group. The expression levels of HSP70 and IgM genes decreased significantly after the addition of total licorice flavonoids to the diet. We speculated that *S. agalactiae* infection stimulated the synthesis of IgM and HSP70 to conduct immune responses. The total flavonoids of licorice can relieve the damaged liver tissue caused by Streptococcus.

In summary, the current study demonstrated that *S. agalactiae* can cause liver injury, which may be related to oxidative stress and abnormal lipid metabolism in tilapia liver tissue. GTFs have a good protective effect on tilapia liver injury caused by *S. agalactiae*, and can repair the damaged antioxidant enzyme system and immune system, alleviating liver tissue damage caused by *S. agalactiae*. Our results highlight the potential for using a Chinese herbal medicine to streptococcal disease in tilapia, and the specific protective mechanism of GTF needs to be further studied.

## Figures and Tables

**Table 1:** The primer sequences used in the present study.

Type	Gene	Primer Sequence (5′–3′)	GenBank Number/References
Fatty acid β-oxidation	*PPAR-α*	F: CTGATAAAGCTTCGGGCTTCCA	NM_001290066.1
	*CPT-1*	R: CGCTCACACTTATCATACTCCAGCT F: TTTCCAGGCCTCCTTACCCA	XM_013268638.3
		R: TGTACTGCTCATTGTCCAGCAGA	
	*ACO-1*	F: GGTCAAAGGCAACAATCAGGAG	NM_001290199.1
		R: GACTCTGCCAAAGGCAACCA	
Lipid droplet formation	*Cideb*	F: CTACCCGACAACGTCATGCT	XM_013266878.3
		R: CCTTGAAATGTGGCCTGCAC	
	*Cidec*	F: TGGAGCCCACATCCTTACAA	XM_005450237.4
		R: TTTTTGGCAGCATAACAGCG	
Fatty acid synthesis	*ACC1*	F: GCGGTGTTCGGCTTGTTTTT	XM_025910662.1
		R: CAAGTCCACCTTCCCTTGGT	
	*FAS*	F: TTTGAGATGTGCTCACAGCTGCA	XM_003454056.5
		R: TCAGCCAGTGAGCTGTGGATGAT	
Fatty acid uptake	*LDLR*	F: TACGGCTTACCAGTCCTCCA	XM_003443172.5
		R: AGGTGACTGGAGCTTGTGTG	
Detoxification	*HSP70*	F:ATTTCAGACGGAGGGAAGCC	XM_019357557.1
		R:CAGCGTTGGACACCTTTTGG	
Immune	*IgM*	F: ACCGAATCGAAAAATGCGGC	KJ676389.1
		R: AACACAACCAGGACATTGGTTC	
Internal reference	*β-actin*	F:CCTGAGCGTAAATACTCCGTCTG	KJ126772.1
		R:AAGCACTTGCGGTGGACGAT	

**Table 2:** Effects of dietary GTF level on growth indices of tilapia.

Index	Dietary GTF Level/(g/kg)			
	0	0.1	0.5	1.0
Initial weight/g	20.00 ± 0.57	19.33 ± 0.88	20.67 ± 0.33	19.67 ± 0.67
Final weight/g	80.19 ± 1.79	82.34 ± 2.12	85.16 ± 2.05	85.90 ± 2.59
WGR, %	3.02 ± 0.21	3.29 ± 0.30	3.12 ± 0.05	3.38 ± 0.20
SGR/(%/d)	1.00 ± 0.04	1.05 ± 0.05	1.07 ± 0.03	1.10 ± 0.04

## Data Availability

Data is contained within the article.
